# The Transtheoretical Model: Is It Still the Best We Have?

**DOI:** 10.2196/75579

**Published:** 2025-07-07

**Authors:** Kaiyuan Cen, Juanyu Lin

**Affiliations:** 1 Cardiovascular Department, Guidong People’s Hospital of Guangxi Zhuang Autonomous Region, Xijiang 4th Road, Wuzhou, 543200, China, 1 86 02460072; 2 Faculty of Medicine and Health Sciences, Universiti of Malaysia Sabah, Kota Kinabalu, Malaysia; 3 Laboratory Medicine Department, GongRen Hospital of WuZhou, Wuzhou, China; 4 Laboratory Medicine Department, The Seventh Affiliated Hospital of Guangxi Medical University, Wuzhou, China

**Keywords:** mhealth, cardiac rehabilitation, mobile health intervention, behavior change, chronic disease management, mobile phone app, smartphone, lifestyle, mobile health

Lunde et al [1] present a thoughtful 5-year follow-up of an app-based intervention following cardiac rehabilitation. The most telling result is not simply that the intervention’s effects diminished over time, but that this decline began as early as 1 year. That timing raises a deeper concern about the relationship between how we design interventions and the behavioral models we continue to use.

The authors structured their intervention using the Transtheoretical Model, a well-known framework in behavior change science. While the model offers a clear sequence of stages, it often fails to reflect how people actually change, especially when living with chronic conditions. Patients do not progress neatly from contemplation to maintenance.—their motivation fluctuates. They pause, regress, and adapt based on circumstances. The idea that someone reaches a stable “termination” phase of change may work in theory, but it rarely holds in practice, particularly after structured support is withdrawn. To their credit, the authors acknowledge that reaching the model’s endpoint within a year is unlikely. However, this admission prompts a more fundamental question: If the endpoint lies beyond the duration of the intervention, then is the model conceptually aligned with what the intervention is trying to achieve? When timelines and theoretical frameworks pull in different directions, the expectations we place on interventions may become difficult to justify. Other models may offer a better fit for this type of long-term digital support. Established frameworks such as Self-Determination Theory and Social Cognitive Theory place more emphasis on sustained motivation, perceived autonomy, and environmental reinforcement [2-5]. These frameworks do not assume that change unfolds in fixed stages. Instead, they treat behavioral maintenance as something that must be supported continuously. This perspective may be more appropriate for post–cardiac rehabilitation populations, where setbacks are common and the need for external structure does not disappear after 1 year.

What makes this study particularly valuable is not only its extended follow-up, but the opportunity it offers to revisit long-standing assumptions in mobile health (mHealth) intervention design. The choice of a behavioral model is not a technical detail. It defines the scope of the intervention, the outcomes we expect, and how we interpret success. The Transtheoretical Model has shaped much of our past work, but it may no longer be flexible enough to guide digital health strategies aimed at long-term behavior change. It is worth asking whether our theories need to evolve in order to keep pace with the people and technologies they are meant to serve.

## Abbreviations

mHealth: mobile health
